# ‘The newest vital sign among pregnant women attending women wellness and research Centre in Qatar: a cross-sectional study’

**DOI:** 10.1186/s12884-021-03542-w

**Published:** 2021-01-21

**Authors:** Sarah Naja, Rowaida Elyamani, Abdullah Al Ibrahim, Noora Al Kubaisi, Rayan Itani, Palli AbdulRouf

**Affiliations:** 1 Department of Community Medicine, Hamad Medical Corporation, P.O. Box 3050, Doha, Qatar; 2 Department of Obstetrics and Gynecology, Hamad Medical Corporation, Doha, Qatar; 3 Primary Health Care Corporation, Doha, Qatar

**Keywords:** Health literacy, Health promotion, Uncontrolled glycaemic level

## Abstract

**Background:**

Health literacy is a vital strategy to consider when designing health-promoting programs, and health literacy is a priority in Qatar’s national health agenda. In the context of pregnancy, inadequate health literacy has been linked to several adverse outcomes among pregnant women such as unplanned conception, smoking, and lack of multi-vitamin intake. Given the paucity of data, this study aimed to assess the level of health literacy and its determinants among pregnant women in the State of Qatar.

**Methods:**

An analytical cross-sectional design was utilized. First, we piloted the measurement tools on 10% of the calculated sample size. Accordingly, the items of the measurement tools were revised. Next, we utilized a structured questionnaire to interview the participants about their socio-demographic characteristics, pregnancy-related factors, and the Newest Vital Sign Tool. A chi-square test was employed to investigate the association level among variables, with significance set to *P* < 0.05. A logistic regression model was used to identify the factors associated with a low literacy level.

**Results:**

We found that almost four in 10 pregnant women (*n* = 138,45.4%) had inadequate health literacy. Furthermore, the insufficient level of health literacy was significantly associated with low educational background, decreased household income, and primigravida. However, uncontrolled glycaemia was the only significant predictor of inadequate health literacy through logistic regression. The scale was found to be reliable, with a calculated Cronbach’s alpha of 0.8.

**Conclusions:**

Low health literacy is common among pregnant women in the State of Qatar. Thus, public health officials should focus on delivering tailored health literacy interventions to pregnant women in the country.

**Supplementary Information:**

The online version contains supplementary material available at 10.1186/s12884-021-03542-w.

## Background

The World Health Organization (WHO) recommends health literacy as a fundamental strategy for achieving several critical targets in the Sustainable Development Goals (SDGs). Hence, several countries worldwide have prioritized health literacy in their policies and practices, including Qatar [1, 2].

Health literacy meanings have expanded in scope and depth. The disagreement led to a variation in central constructs of health literacy identified by a group of academics as a set of skills; others focused on knowledge, and still others describe it as a hierarchy of function [3]. For instance, the WHO defines health literacy as ‘The degree to which people can access, understand, appraise and communicate information to engage with the demands of different health contexts to promote and maintain good health across the life-course’ [2]. However, the more commonly used definition in recent literature is presented by the Institute of Medicine (IOM) as ‘The degree to which individuals can obtain, process, and understand basic health information and services needed to make appropriate health decisions’ [4, 5]. Both definitions’ treat a person’s skills as core to health literacy [6].

**Fig. 1:**
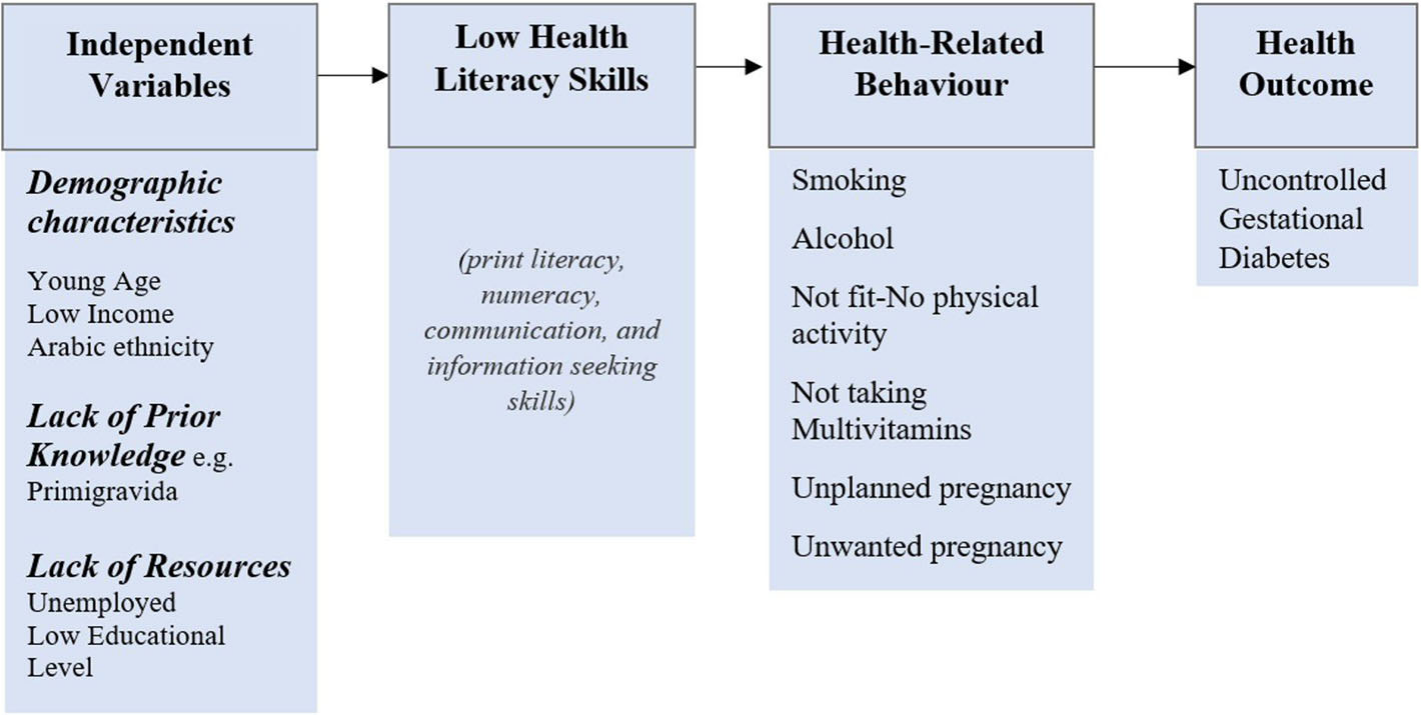
Conceptual Framework of Health Literacy Skills in the Context of Pregnancy

Despite the extensive studies in this area over the past decades, the consensus on a standard conceptual framework that can serve various contexts remains debatable with up to 12 concepts that allow investigators to answer their research question [3]. Individual-level health literacy models are commonly used. However, these models have been criticized for being static and less dynamic [6]. For that, we adapted the continuum theoretical framework of health literacy to be applied in the context of pregnancy [7]. It is a dynamic individual-level model that investigates health literacy through two pathways: The first pathway leads to the development of inadequate individual’s health literacy skills, and the second pathway mediates the effects of health literacy on development of adverse health outcomes during pregnancy [7, 8]. The adapted health literacy skills (HLS) framework is shown in Fig. 1.

A woman’s ability to obtain, process, understand, and communicate about health-related information needed to make informed health decisions is critical to both mother and foetus. To illustrate, an earlier systematic review on health literacy and reproductive health that limited health literacy was linked to the lack of prenatal care, prenatal vitamins, worse pregnancy outcomes, and may predict smoking relapse and depression [9–11]. Additionally, health literacy among pregnant women was identified as the only modifiable factor for breastfeeding self-efficacy [12]. Consequently, pregnant women with low health literacy were significantly more likely to have an unplanned pregnancy and lack access to a healthcare provider, reflecting negatively on their pregnancy outcomes [13]. Thus, a higher level of health literacy among pregnant women translates into a healthier pregnancy [14, 15].

A few studies have investigated the level of health literacy among pregnant women. In south Iran, a country that shares a similar social and cultural context in the gulf region - an investigation uncovered that nearly one-sixth (15.5%) of pregnant women had an inadequate health literacy level. Fewer than half (41.7%) reported a border-line level of health literacy [16]. Another cross-sectional survey of pregnant women in Ireland revealed a higher percentage of limited health literacy. Furthermore, the study identified one in every four participants (25.3%) as having limited health literacy [17]. Several factors were associated with low health literacy, including a low educational level and household income [16, 17].

Given the significant burden of low health literacy on pregnant women, the Qatar National Strategy prioritizes healthy pregnancy and emphasizes the importance of health literacy and identifying and addressing this modifiable risk factor to improve health outcomes. Women comprise about a quarter (24.4%) of the population (1,699,435) in Qatar— more than half of which (59.8%) are of the reproductive age group (15–44 years old) [1, 18]. However, health literacy in the context of pregnancy has not been investigated in Qatar and the Gulf region.

### Purpose

The primary purpose of this study was to examine the level of health literacy among pregnant women attending a specialized obstetrics and gynaecology hospital (Women’s Wellness and Research Centre, WWRC) in Qatar. The secondary objective was to investigate the determinants associated with inadequate health literacy and its related health outcome.

## Methods

### Study design and setting

This study was an analytical cross-sectional study. It involved pregnant women attending the antenatal clinics of Women’s Wellness and Research Centre’ (WWRC) in Qatar between October and December of 2019. The WWRC is a governmental health facility located in Doha (capital of Qatar). It is a major hospital that provides outpatient and inpatient services for women during their reproductive age and accommodates 17,000 births per year. The WWRC serves a broad segment of the country’s population with different economic, educational, cultural, and social backgrounds. In contrast to private hospitals that mainly cater to clients of a high socioeconomic class, WWRC’s patient cohort offers a good representation of the whole community. The WWRC also offers antenatal health services to all pregnant women at a subsidized rate and is accessible by self-referral or referral from a primary health care provider. The antenatal care package includes providing educational classes on a healthy pregnancy (healthy eating, exercise, and family planning) by an expert team of nurses and midwives. The expecting mothers are also followed by obstetricians through periodic prenatal consultations in seven outpatient clinics. The average number of patients attending each clinic can reach 20 per shift (morning or evening). WWRC’s antenatal clinic participation rate was as high as 70% of the total live births during 2019 (about 12,896 pregnant women) [19].

### Study population and sampling technique

The target population comprised any pregnant woman of reproductive age (18–49 years) who was willing to participate and attending WWRC’s outpatient clinics, regardless of trimester, and capable of communicating in English or Arabic.

A non-probability (convenient) sampling technique was employed to enrol participants in this study. First, the researchers obtained the total number of pregnant women attending WWRC’s antenatal clinics. Secondly, trained data collectors attended the outpatient clinics and informed the potential participant about the study. The expectant mothers were screened for eligibility and asked to sign a consent form if willing to participate. This process continued until the calculated sample size was fulfilled.

### Sample size and enrolment of participants

To the best of our knowledge, no previous research has yet studied health literacy among pregnant women in Qatar. Thus, the researchers utilized an effect size of 50%, a 5% degree of precision, 95% confidence limits, and a design effect of 1. Accordingly, the sample size was calculated from the following formula: n = [Z2 1- /2 x p x (1-p)] / d2 X Design Effect [20].

Eligible participants included all pregnant women of reproductive age (18–49) years old who were willing to participate and fulfil the selected eligibility criteria. The inclusion criteria included Qatari and non-Qatari nationalities, participants with verified pregnancy [laboratory assessment of human chorionic gonadotropin (HCG) in urine or blood, and ultrasound confirmation of viable foetus] [21]. Sufficient knowledge of English and/or Arabic was needed to cooperate with data collection procedure. All subjects gave informed consent and permission as well as we did not have specific exclusion criteria.

### Data collection

After securing consent (Supplementary file 1), the data collector interviewed the participants in a private area near the antenatal clinics. Each interview took an average of 10 min for each participant. The data collector then took the anthropometric measures for those in their first trimester. Next, the data collector reviewed the pregnancy-related notebook (a medical record) of the participant for the body mass index (BMI), medical history, medications, and laboratory results. After completing the interviewer-guided questionnaire, the participants were given an ice cream nutrition label and asked a series of six questions from the ‘Newest Vital Sign (NVS)’ tool. Each respondent had to refer to the ice cream label while answering these verbal questions. The data collector recorded the responses on a score sheet, which contained the correct answers. Accordingly, the data collector assessed the participant’s health literacy level.

### Variables and measures

The study’s dependent variable corresponds to the level of health literacy among pregnant women. Health literacy measurement is a challenge especially since health literacy’s conceptual definition is continuously evolving and reflects negatively on the accuracy and construct validity of the operational tools. The most-reported tools to use among pregnant women across literature are Rapid Estimate of Adult Literacy in Medicine (REALM) and Test of Functional Health Literacy in Adults (TOFLA). However, one significant limitation of these tools is their focus on reading proficiency rather than capturing health literacy’s health promotion. We assessed the dependent variable through the standard measurement tool ‘The Newest Vital Sign.’ The instrument measures multiple aspects of print literacy, numeracy, and oral literacy (i.e., communication skills). It offers quantitative assessment of a health literacy dynamic construct [22].

The Newest Vital Sign was developed in English in the United States. The English Version was tested for its psychometric properties among the same population (pregnant women) and showed acceptable validity compared to another tool (TOFLA) at a cut-off score of four [23]. The Newest Vital Sign has also been revealed to be a reliable and valid instrument in Spanish, Portuguese, and Dutch [24–26]. The ‘Newest Vital Sign’ was translated and adapted to the Arabic language and tested for cultural suitability [27, 28]. It is one of the most frequently adapted tools to assess health literacy in the Eastern Mediterranean Region [29].

The test requires only 3 min for administration. It includes six questions related to the ice-cream nutrition label; the answers are binary (Yes, No). Each correct explanation of the NVS items is one point, and the total score is the summation of the total points of the six items. When the total score lies between 0 and 1, it indicates ‘high likelihood of limited health literacy,’ 2–3 ‘possible limited health literacy,’ and 4–6 means ‘adequate health literacy’. In the end, we dichotomized the level of health literacy into inadequate health literacy (< 4), adequate health literacy (> 4). The English and Arabic versions were reliable measurement tools and provided an acceptable level of internal consistency (Cronbach alpha> 0.76) [30, 31].

Upon the adapted Conceptual Framework, the independent variables interact based on three categories (Moderators, Mediators, and Health Outcome):

1. Moderators: Stated as variables that lead to health literacy development mainly including socio-demographic characteristics (age, nationality, education, occupation, family size), pregnancy-related characteristics (gravidity, number of children, and gestational age).
2. Mediators: The variables that influence the relationship between health literacy and health outcome, behavioural factors (smoking, alcohol use, and physical activity).
3. Health outcomes: The acute illnesses including gestational diabetes and uncontrolled glycaemic level HBA1C > 6.5%.

We assessed the independent variables through structured and interviewer-guided questionnaires included in closed-ended questions on the socio-demographic characteristics (age, nationality, education, occupation, family size), pregnancy-related characteristics (gravidity, number of children, gestational age, desire for pregnancy, acute illnesses including gestational diabetes, uncontrolled glycaemic level HBA1C > 6.5%, chronic illnesses), and behavioural factors (smoking, alcohol use, and physical activity). We evaluated fitness through Readiness Medical Examination (PARMed-X). The fitness tool embraces exercise and other activities such as playing, running, walking, and recreational activities, but it excludes housework. Unfit participants were those who perform physical activity at a rate less than once or twice a week for fewer than 20 min (index zero) while active pregnant women practice physical activity once to twice a week for 20 min or more than twice a week for less than 20 min (index one). Finally, pregnant women who perform a physical activity more than twice a week for more than 20 min were considered fit (index two). To sum up, three categorical variables were calculated based on physical activity index score: Index 2(Fit), Index 1 (Active), and Index 0 (Not fit) (see Supplementary file 2) [32].

An expert panel established the questionnaire’s face validity (English and Arabic versions) and its relevance to the study objectives. Additionally, the content validity was verified through an extensive literature review to ensure the consistency of the contents and scale level. Using Lawshe’s method, each item was rated for its importance and relevance by applying a three-point scale: (1) not necessary, (2) useful but not essential, and (3) essential. The universal agreement between the three evaluators was 80%.

We conducted piloting on 10% of the total sample. Piloting ensured pre-testing for any difficulties and any inappropriateness of the tool. We measured the time needed to complete each questionnaire and the proper accompanying logistics that maintain the participants’ privacy and anonymity. We made the appropriate changes to the interviewer-guided questionnaire and excluded the pilot sample from analyses.

### Analysis

We analysed data through IBM SPSS for Windows version 22. The statistical analysis involved descriptive summarization of the variables: Data were presented in tables in frequency and percentages for categorical variables and mean ± standard deviation (SD) for continuous variables. The Kolmogorov test and Shapiro Wilk test were employed to test the normality of distribution of the dependent variable (NVS scores-continuous variable) followed by bootstrapping.

We conducted a bivariate analysis to test for associations between dependent and independent variables through Pearson’s chi-squared test (odds ratio (OR) and 95% CI). We then included all significant determinants in multivariable logistic regression analysis and computed adjusted odds ratio (entry method Logistic Regression). We also performed reliability testing and principal component analysis of the ‘Newest Vital Sign’ tool. The significance level was set at *p* < 0.05. In the end, we performed an audit on 10% of the data entered by another researcher to ensure the quality of the data entered.

## Results

### Participants’ characteristics

We approached 320 pregnant women during this period to participate in the study. After excluding the pilot sample, the total number of pregnant women included was 304 participants.

The age of the enrolled participants averaged 30 years (SD + 3.9) and ranged between 20 and 39 years. Most of the respondents were expatriates (74%), and nearly half did not attend university (47.4%). Few were alcoholics (0.7%) and smokers (5.9%). Most of the pregnant women were in the 2nd trimester (63.1%), multigravida (64.3%), and had gestational diabetes (33.5%). Diabetic participants did not receive insulin or any other hypoglycaemic medications.

### Sample distribution of health literacy

The health literacy distribution showed not a perfectly symmetrical distribution-mildly asymmetrical to the left. Scores ranged from [0–14], with a mode of 6 and a median of 5.

The calculated mean was 3.45, 95% CI [3.16–3.72], SD + 2.5, and standard error 0.14. The skewness coefficient was − 0.05 with a kurtosis coefficient of − 0.70 (less than zero), indicating light tail (platykurtic distribution).

The statistical bootstrapping method showed that the mean’s actual value is the same as that without bootstrapping 3.45, 95% CI [3.16–3.72].

### Prevalence of health literacy

Of the 304 participants, almost half (54.6%) showed adequate health literacy, and a third (32.9%) demonstrated a high likelihood of limited health literacy (**Table** 1**)**.

**Table 1:** The distribution of false answers on the Newest Vital Sign tool among pregnant women attending Women’s Wellness and Research Centre in Qatar, 2019 (*N* = 304)**.**

Newest Vital Sign Questions	False Answers	
***N*** = 304	Frequency (n)	Percentage (%)
1. How many calories (kcal) will you eat if you eat the whole container?	**130**	**42.8**
2. If you are advised to eat no more than 60 g of carbohydrate for dessert, what is the maximum amount of ice cream you could have?	**158**	**52**
3. Imagine that your doctor advises you to reduce the amount of saturated fat in your diet. You usually have 42 g of saturated fat each day, some of which comes from one serving of ice cream. If you stop eating ice cream, how many grams of saturated fat would you be eating each day?	**99**	**32.6**
4. If you usually eat 2500 cal each day, what percentage of your daily calorie (kcal) intake will you get if you eat one serving of ice cream?	**132**	**43.4**
5. Is it safe for you to eat this ice cream?	**133**	**43.8**
6. Why not?	**133**	**33.8**

### Determinants of health literacy

The result showed that low educational levels, low household income, and unemployment are statistically associated with an inadequate health literacy level. Nationality and age did not show any significant association (Table 2).

**Table 2:** Moderators: Demographical and lack of resources characteristics and its associations with the level of literacy among the participants attending Women’s Wellness and Research Centre in Qatar, 2019 (*N* = 304).

Socio-demographic characteristics	Total		Health Literacy Level					
			Inadequate (< score 4)		Adequate (**>**score 4)			
	***N*** = 304	(%)	n	(%)	n	(%)	χ^**2**^	OR,95%CI
**Age (years)**								
[20–24]	24	(7.9)	15	(62.5)	9	(37.5)		
[25–29]	105	(34.5)	49	(46.7)	56	(53.3)		
[30–34]	129	(42.4)	57	(44.2)	72	(55.8)	**3.2**	**–**
[35–39]	46	(15.1)	17	(37.0)	29	(63.0)		
Mean + SD [30 + 3.9] Range [20–39]								
**Nationality**								
Qatari	79	(26)	35	(44.3)	44	(55.7)	**0.01**	1.0 [0.6–1.7]
Non-Qatari	225	(74)	103	(45.8)	122	(54.2)		
**Ethnicity**								
Arabs	298	(98)	137	(46.6)	161	(54.4)	**0.38**^**a**^	0.2 [0.02–2]
Non-Arabs	6	(2)	1	(16.7)	5	(83.3)		
**Educational level**								
Secondary*	144	(47.4)	75	(52.1)	69	(47.9)	**4.7**	1.6 [1.1–2.6]
University	160	(52.6)	63	(39.4)	97	(60.6)		
**Occupation**								
Housewife*	140	(46.1)	71	(50.7)	69	(49.3)	**4.0**	1.6 [1.1–2.5]
Working	164	(53.9)	67	(40.9)	97	(59.1)		
**Household income (QR)**								
< 10,000 *	1	(0.3)	0	(0)	1	(100)		**–**
10,001–20,000	160	(52.6)	58	(53.1)	75	(46.9)	**8.1**	
< 20,001	143	(47)	53	(37.1)	90	(62.9)		

*n* number of observations in the sample*, N* population inference based on weights and sampling design*; %* estimated prevalence based on weighted frequencies*, QR* Qatari Riyal*, OR* Odd Ratio; *p value *<* *0.05; a* Fisher test*, χ*^*2*^ Chi-square

Table 3 shows that being primigravida, having less than two children, and uncontrolled glycaemic control are statistically associated with an inadequate health literacy level. However, gestational age, unplanned pregnancies, alcohol use, and smoking did show any significant association.

**Table 3:** Clinical and behavioural characteristics and its associations with the level of literacy among the of the participants attending Women’s Wellness and Research Centre in Qatar, 2019 (*N* = 304).

Clinical and behavioural characteristics	Total		Health Literacy Level					
			Inadequate (< score 4)		Adequate (**>**score 4)			
	***N*** = 304	(%)	***n***	(%)	***n***	(%)	χ^**2**^	OR, 95%CI
**Gravida**								
Primigravida	80	(35.7)	44	(55.0)	36	(45.0)		0.5 [0.3–0.9]
Multigravida*	224	(64.3)	94	(42.0)	139	(58.0)	**3.5**	
**Trimesters**								
First	6	(1.7)	5	(83.3)	1	(16.7)		
Second	192	(63.1)	86	(44.8)	106	(55.2)	**3.6**	**–**
Third	107	(35.2)	48	(44.9)	59	(55.1)		
**Number of Children**								
< 2	85	(38.8)	111	(50.7)	108	(49.3)	**7.1**	0.4 [0.2–0.7]
> 2*	219	(61.2)	27	(31.8)	58	(68.2)		
Mean + SD [2+ 1]								
Range [0–5]								
**Glycaemic index**								
Uncontrolled	83	(27.3)	76	(91.6)	7	(8.4)	**98**	0.03 [0.01–0.08]
Controlled*	221	(72.7)	62	(28.1)	159	(71.9)		
**Planned pregnancy**								
Yes	230	(75.6)	109	(47.4)	121	(52.6)	**1**	0.7 [0.4–1.2]
No	74	(24.3)	29	(39.2)	47	(60.8)		
**Fitness**								
Unfit	250	(82.2)	110	(44.0)	140	(56)		
Active	35	(11.5)	19	(54.3)	16	(45.7)	**1.8**	**–**
Fit	19	(6.25)	9	(47.4)	10	(52.6)		
**BMI**								
Underweight [< 18.5]	18	(6.0)	8	(44.4)	10	(55.6)		**–**
Normal [18.5–24.9]	211	(69.4)	100	(47.4)	111	(52.6)	**2.9**	
Overweight [25–29.9]	69	(22.6)	28	(40.6)	41	(59.4)		
Obese > 30	6	(2.0)	22	(33.3)	4	(66.7)		

*n =* number of observations in the sample*, N* population inference based on weights and sampling design*, %* estimated prevalence based on weighted frequencies*, OR* Odd Ratio, *p value *<* *0.05, χ*^*2*^ Chi-square

Upon logistic regression analyses, an uncontrolled glycaemic level was predictive of inadequate health literacy. Other variables failed to show a significant association (Table 4).

**Table 4:** Logistic regression of the predictors of low health literacy among pregnant women (*n* = 800).

Variables	Variables in the equation Health Literacy						
	B	S. E	Wald	Sig.	aOR	95% CI for aOR	
						Lower	Upper
**Education**							
Low education	0.3	0.29	1.07	0.30	1.36	0.76	2.40
High education					1		
**Occupation**							
Housewife	0.15	0.29	0.28	0.57	1.17	0.65	2.08
Working					1		
**Household income**							
Low income	0.31	0.29	1.12	0.29	1.35	0.76	2.4
High income					1		
**Gravidity**							
Primigravida	0.42	0.33	1.61	0.20	1.53	0.79	2.95
Multigravida					1		
**Number of children**							
< 2	0.58	0.35	2.63	0.10	1.78	0.88	3.58
> 2					1		
**Glycaemic level**							
Controlled *	−3.35	0.43	60.1	0.0001*	0.03	0.01	0.08
Uncontrolled					1		

*B:* B coefficient*; aOR* adjusted Odd Ratio, *P value *<* *0.05, CI* Confidence Interval.

The model was obtained using entry selection. The final model was adjusted for age, nationality, trimester, BMI, unplanned pregnancy.

### Principal component and reliability testing

The scale shows Cronbach’s alpha of 0. 86 and the first four items explained up to 65% of the variance.

## Discussion

This study investigated the level of health literacy among pregnant women visiting the antenatal clinics of WWRC in Qatar. Almost one-third of participants (32.9%) demonstrated a high likelihood of limited health literacy. A significant association was found between inadequate health literacy and a low level of education, low income, unemployment, primigravida, having less than two children, and an uncontrolled glycaemic level.

The prevalence of inadequate health literacy in our study was higher than that of an Irish study (25.3%) [17]. The comparison is relevant as both studies utilized the tool ‘Newest Vital sign’ tool. This discrepancy in health literacy level between Ireland and Qatar could be due to the cultural and multinational differences between these countries [29].

Similarly, other studies in Iran and the United States identified nearly one-sixth (15.5%) and one-fifth (22%) of participants to have limited health literacy based on the Test of Functional Health Literacy in Adults (TOFLA**)** [13, 16]. However, the TOFLA instrument fails to capture the health promotion-related aspect of health literacy as stated in an earlier systematic review [22].

In contrast to our study, Iranian research indicated that older pregnant women had a significantly lower health literacy levels than younger mothers. Moreover, the investigators argued that age could be a confounding factor for education level, which was lower among older Iranian women. These women’s occupation status was significantly associated with higher health literacy levels because they had a better socioeconomic status [16], which is consistent with our study where almost half (46%) of the participants were housewives.

Nevertheless, our results indicated that pregnant women with a higher education level and better household income possessed a higher level of health literacy. This result is consistent with previous papers published in the Czech Republic [14], Iran [16], Ireland [17], Turkey [33], and the United States [34].

These studies identified a significant association between low socio-demographic factors (low income, low educational level) and low health literacy levels. Hence, tackling these factors could help in improving health literacy and will enhance the patient’s ability to obtain information that would guide their health-related decisions. In addition, high health literacy was significantly associated with multigravida and having more than two kids. The knowledge and awareness could be from their previous pregnancies [14].

Family planning behaviour (unplanned pregnancy) and lack of multi-vitamins intake were not statistically linked to a low health literacy level. This result contrasts that of an earlier systematic review [9]. This association was not significant in our population due to the confounding effect of medical advice on pregnant women and the importance of taking multi-vitamins and family planning behaviour.

### Strengths and limitations

This research is the first to examine health literacy status among pregnant women in the Arab Gulf region. The regional body of research has focused on adult populations and revealed some gender differences.

Statistical analysis requires inferencing, and uncertainty can be dangerous. Here, we draw additional samples (with replacement) bootstrap from sample itself. Thus, the results of our study can be generalizable to the population as a whole. Furthermore, several strategies were employed to decrease the measurement bias, included selecting a reliable tool (NVS) with good internal consistency (Cronbach’s alpha = 0.866) that had been previously validated in the literature [23]. Similarly, the content and face validity of the questionnaire were assured through appropriate techniques. A logistic regression analysis was pursued to avoid any confounder effect in this study.

Nevertheless, this was a cross-sectional study that lacked the temporality between variables and compromised any causation effect. Utilizing a non-probability sampling technique might also undermine the external validity of the study. However, the sample’s heterogenicity encompassed various socio-demographic subgroups and should, on average, provide an accurate account of the population [35].

## Conclusion

This study indicates that low health literacy is common among pregnant women attending the antenatal clinics of the WWRC in Qatar. Health officials should design and implement targeted interventions to promote health literacy among pregnant women with a low educational level as well as low-income, unemployed, and primigravida subjects. This strategy is a fundamental element in improving health literacy level, which improves the clinical outcomes of pregnancy for both mother and foetus.

## Supplementary Information

**Additional file 1.** Written consent form English-language copy.

**Additional file 2.** Questionnaire English-language copy.

## Data Availability

The datasets used and/or analysed during the current study are available from the corresponding author on reasonable request.

## Abbreviations

WHO: World Health Organization
SDGs: Sustainable Development Goals
REALM: Rapid Estimate of Adult Literacy in Medicine
TOFLA: Test of Functional Health Literacy in Adults
NVS: Newest Vital Sign
BMI: Body Mass Index
OR: Odd Ratio
CI: Confidence Interval
