# Supplementary material for: ‘The newest vital sign among pregnant women attending women wellness and research Centre in Qatar: a cross-sectional study’
Source: BMC Pregnancy Childbirth. 2021 Jan 21;21:73. doi: 10.1186/s12884-021-03542-w (PMC7819321; doi:10.1186/s12884-021-03542-w)
Supplement: Supplementary file 1 — Additional file 1. Written consent form English-language copy. [file 12884_2021_3542_MOESM1_ESM.pdf]

RESEARCH CONSENT FORM

1. Title of research

Health Literacy among Pregnant Women in Qatar 2019: The Newest Vital Sign

2. Principal Investigator

Sarah Naja –Community Medicine Resident at Hamad General Hospital

3. Why are we inviting you to join this research?

The investigator is conducting this research at the Women Research Center.

We are inviting you to join because you are pregnant attending in antenatal clinic at Women Research Center and your health literacy is important to us as it may affect you and your baby.

4. What should you know about this research?

- We will explain the research to you
- Whether or not you join is your decision (you can accept or refuse no matter who is inviting you to participate)
- Please feel free to ask questions or mention concerns before deciding, or during or after the research
- You can say yes but change your mind later
- We will not hold your decision against you

5. Who can you talk to?

If you have questions or concerns, or if you think the research has hurt you, talk to the research team at:
Dr. Sarah Naja (Community medicine Resident at Hamad General Hospital) phone number: 33615489
If you have questions about your rights as a volunteer, or you want to talk to someone outside the research team, please contact:

- HMC Institutional Review Board (HMC-IRB) Chair at 5554 6316
- HMC-IRB Office at 4025 6410 (from Sunday to Thursday between 7:00am-3:00pm) or email at irb@hamad.qa

6. Why are we doing the research?

Our goal in carrying out this research is to find out the level of health literacy and to identify if there is relevant associated factors with low level of literacy .We hope that we will be able to identify the common factors leading to low health literacy and this can help us in guiding stalk holders in formulating healthcare related programs.

7. How long will the research take?

Institutional Review Board · Hamad Medical Corporation · Doha · Qatar

MRC-01-18-188 Validity: 08 05 2019 - 06 05 2020 E-stamped 12 Jun 2019

RESEARCH CONSENT FORM

**1. Title of research**
Health Literacy among Pregnant Women in Qatar 2019: The Newest Vital Sign
**2. Principal Investigator**
Sarah Naja –Community Medicine Resident at Hamad General Hospital
**3. Why are we inviting you to join this research?**
The investigator is conducting this research at the Women Research Center.<br><br>We are inviting you to join because you are pregnant attending in antenatal clinic at Women Research Center and your health literacy is important to us as it may affect you and your baby.
**4. What should you know about this research?**
• We will explain the research to you<br>• Whether or not you join is your decision (you can accept or refuse no matter who is inviting you to participate)<br>• Please feel free to ask questions or mention concerns before deciding, or during or after the research<br>• You can say yes but change your mind later<br>• We will not hold your decision against you
**5. Who can you talk to?**
If you have questions or concerns, or if you think the research has hurt you, talk to the research team at:<br>Dr. Sarah Naja (Community medicine Resident at Hamad General Hospital) phone number: 33615489<br>If you have questions about your rights as a volunteer, or you want to talk to someone outside the research team, please contact:<br>• HMC Institutional Review Board (HMC-IRB) Chair at 5554 6316<br>• HMC-IRB Office at 4025 6410 (from Sunday to Thursday between 7:00am-3:00pm) or email at irb@hamad.qa
**6. Why are we doing the research?**
Our goal in carrying out this research is to find out the level of health literacy and to identify if there is relevant associated factors with low level of literacy .We hope that we will be able to identify the common factors leading to low health literacy and this can help us in guiding stalk holders in formulating healthcare related programs.
**7. How long will the research take?**

MRC-01-18-188 Validity: 08 05 2019 - 06 05 2020 E-stamped 12 Jun 2019

You will be seen one time for completing the questionnaire and no follow up (cross sectional). The time to complete questionnaire may take 10 min.

We expect the research to last for 1month for data collection.

8. How many people will take part?

We plan to study people (n= 320) pregnant women at Women Research Center.

9. What happens if you take part?

If you agree to join, we will ask you to do the following:

When you visit your doctor or attend the clinic, you will still receive all your usual care, laboratory tests and treatment. What is different is that we will take a little more of your time to ask you some questions which we have put together in a face-face interview questionnaire and self-administrated tool. This should take around 10 minutes to complete.
We will ask you about your feelings and factors that could be related to it. You will not have to pay anything extra for this research. You will however be charged normally for your routine consultation and tests.

10. Could the research be bad for you?

This study involves asking you questions about health literacy level, nothing intrusive . However please be assured that all information you provide will be treated sensitively and in strict confidence. It will not be shared with anyone outside of the research or be made available except for use in this research. You will not also be identified by name at any stage in the research.

11. Could the research be good for you?

Some individuals may find addressing health literacy an educational opportunity so that they know better about their condition useful and reassuring.
However you may not benefit directly from this particular study. Instead the results can provide lessons for us to plan improvements in treatment and the service.

12. What happens to information about you?

We will make efforts to secure information about you. This includes using a code to identify you in our records instead of using your name. We will not identify you personally in any reports or publications about this research.

Institutional Review Board Hamad Medical Corporation - Doha

MRC-01-18-188 Validity: 08 05 2019 - 06 05 2020 E-stamped 12 Jun 2019

1. We will provide private space for you during consultation and the interview.
2. We will store all information you provide securely and will not make it available to anyone else.

We cannot guarantee complete secrecy, but we will limit access to information about you. Only people who have a need to review information will have access. These people might include:

- Members of the research team who make sure the study is done properly and that your rights and safety are protected.
- Your doctors and nurses

13. What if you don't want to join?

You can say no and we will not hold it against you and you will receive normally your routine care.

14. What if you join but change your mind?

You can stop participating at any time and we will not hold it against you and the data will be discarded.

15. What else should you know?

This research is funded by Women Research Center.
You are free to ask as many questions as you like before, during or after you decide to give consent to participate in this research study. The information in this form is only meant to better inform you of all possible risks or benefits. Your participation in this study is voluntary. You do not have to take part and your refusal to participate will involve no penalty or loss of rights to which you are entitled. The investigator(s) may stop your participation in the study without your consent for reasons such as: it will be in your best interest; you do not follow the agreed study plan; or you experience a study-related adverse effect, discomfort, injury or other unexpected incident.

16. Additional Choices

We would like your permission to contact you about participating in future studies. You may still join this study even if you do not permit future contact. You may also change your mind about this choice. Please initial your choice below:

___________YES, you may contact me

___________NO, you may NOT contact me

Institutional Review Board · Hamad Medical Corporation · Doha - Qatar
Hamad

MRC-01-18-188 Validity: 08 05 2019 - 06 05 2020 E-stamped 12 Jun 2019

Research Consent Form

Signature Page for Capable Adult

Volunteer

I voluntarily agree to join the research described in this form.

____
Printed Name of Volunteer

____
Signature of Volunteer Date

Person Obtaining Consent

I document that:

- *I (or another member of the research team) have fully explained this research to the volunteer.*
- *I have personally evaluated the volunteer's understanding of the research and obtained their voluntary agreement.*

____
Printed Name of Person Obtaining Consent

____
Signature of Person Obtaining Consent Date

Witness (if applicable)

I document that the information in this form (and any other written information) was accurately explained to the volunteer, who appears to have understood and freely given Consent to join the research.

____
Printed Name of Witness

____
Signature of Witness Date

Institutional Review Board · Hamad Medical Corporation · Doha · Qatar
Hamad

MRC-01-18-188 Validity: 08 05 2019 - 06 05 2020 E-stamped 12 Jun 2019
