# Supplementary material for: ‘The newest vital sign among pregnant women attending women wellness and research Centre in Qatar: a cross-sectional study’
Source: BMC Pregnancy Childbirth. 2021 Jan 21;21:73. doi: 10.1186/s12884-021-03542-w (PMC7819321; doi:10.1186/s12884-021-03542-w)
Supplement: Supplementary file 2 — Additional file 2. Questionnaire English-language copy. [file 12884_2021_3542_MOESM2_ESM.pdf]

Participant number………………..

Date of data collection

……../ ……. / 2018

As you are pregnant and your health status matters to us, we would like to ask you some questions. It will include ***face to face*** interview based questionnaire ***(section I)*** and self-administrated tools ***vital sign tool (section II)***. ***It will not take more than 15 minutes.***

- **Section I: Questionnaire**

This is an **interview based** questionnaire, going to investigate some aspects related to socio demographical factors, economic factors, medical history, life style habit. **One answer** must be selected, **unless mentioned** that multiple answers could be picked.

Institutional Review Board Hamad Medical Corporation - Doha - Qatar Hamad

MRC-01-18-188 Validity: 08 05 2019 - 06 05 2020 E-stamped 12 Jun 2019

Participant number………………..

Date of data collection

……../ ……. / 2018

As you are pregnant and your health status matters to us, we would like to ask you some questions. It will include ***face to face*** interview based questionnaire ***(section I)*** and self-administrated tools ***vital sign tool (section II)***. ***It will not take more than 15 minutes.***

- **Section I: Questionnaire**

This is an **interview based** questionnaire, going to investigate some aspects related to socio demographical factors, economic factors, medical history, life style habit. **One answer** must be selected, **unless mentioned** that multiple answers could be picked.

MRC-01-18-188 Validity: 08 05 2019 - 06 05 2020 E-stamped 12 Jun 2019

**Q No.**	Questions	Answers
**A- Socio-demographical factors**		
1.	How old are you? (In full years)	……………………
2.	What is your nationality?	………………..
3.	What is your **highest level** of education?	☐ (3)Illiterate<br>☐ (2)Primary education<br>☐ (1)Secondary education<br>☐ (0)Higher education
4.	You are living alone:	☐ (0)No<br>☐ (1)Yes
5.	How many members are living in your house including yourself? (family size)	……………………
6.	Marital status:	☐ (0)Married<br>☐ (1)Divorced<br>☐ (2)Separated<br>☐ (3)Widowed
**B- Economic factors**		
7.	Employment Status:<br>Are you currently ….	☐ (0)House wife (skip to Q-12)<br>☐ (1)Student(skip to Q-12)<br>☐ (2)Employed
8.	If employed, state your current occupation	....
9.	How many hours you work per week?	…………………..(hours/week)
10.	What is your partner work status?	☐ (0)Employed ☐ (1)un-employed
11.	What is approximately your monthly family income?	☐ (2)Up to 10,000 QR<br>☐ (1)10,001-20,000 QR<br>☐ (0)Equal or more than 20,001 QR

Institutional Review Board Hamad Medical Corporation · Doha · Qatar
Hamad
MRC-01-18-188 Validity: 08 05 2019 - 06 05 2020 E-stamped 12 Jun 2019

12. Your financial status is:
   - ☐ (0)Strongly Sufficient
   - ☐ (1)Sufficient
   - ☐ (2)Not sufficient
   - ☐ (3)Strongly not sufficient

13. Are you facing any financial difficulties?
   - ☐ (3)Always
   - ☐ (2)Sometimes
   - ☐ (1)Rarely
   - ☐ (0)Never

C-History of current pregnancy

14. Is this your first pregnancy?
   - ☐ (0)No
   - ☐ (1)Yes

15. How many children do you have?

   ………….

16. Did you have previous abortion?
   - ☐ (0)No, I don't have abortion.
   - ☐ (1)Yes, I had abortion (<20 week gestation).

17. Did you have previous still birth?
   - ☐ (0)No, I don't have still birth.
   - ☐ (1)Yes, I had still birth (>20 week gestation).

18. For how many weeks are you pregnant?

   ……………… (Weeks.)

19. Did you planned (intended) this pregnancy?
   - ☐ (1)Yes, through stopping contraception or adapting preconception behavior.
   - ☐ (0)No, I was not planning for this pregnancy and I was adapting anti-conception behavior such as using contraception.

20. Do you want this pregnancy (wanted pregnancy)?
   - ☐ (1)Yes, I want this pregnancy so I will continue this pregnancy.
   - ☐ (0)No, I don't want this pregnancy so I wish to have abortion.

21. Did you suffer previously from infertility (inability to conceive spontaneously for more than 1 year)?
   - ☐ (0)No
   - ☐ (1)Yes

22. Currently, how did you conceive?
   - ☐ (0)Spontaneously
   - ☐ (1)Induced, through ovulatory medications
   - ☐ (2)Induced through In vitro fertilisation (IVF)

MRC-01-18-188 Validity: 08 05 2019 - 06 05 2020 E-stamped 12 Jun 2019

23. Have you been **diagnosed** with any of these health problems during your ***current pregnancy***?(Plus medical records will be reviewed)

Single or multiple choices could be selected if applicable

- ☐ Diabetes during pregnancy (GDM)
- ☐ Pregnancy induced Hypertension
- ☐ Pre eclampsia
- ☐ Eclampsia
- ☐ Urinary tract infection
- ☐ Hyperemesis Gravidarium
- ☐ Thyroid disorders
- ☐ Anemia
- ☐ Pregnancy related Anxiety
- ☐ Antenatal Depression
- ☐ Panic attack
- ☐ Schizophrenia
- ☐ Bipolar disorder
- ☐ Abnormal ultrasound for embryo
- ☐ Abnormal ultrasound for placenta or amniotic fluids
- ☐ None
- ☐ Others please specify…..

☐ **Not applicable for non-GDM *N.B: if not GDM shift to Q-31***

D-Lab results related to Gestational Diabetes From medical records

24. Fasting blood glucose level result? (after 8 hours fasting) …………….. (mmol/L)

25. Glucose tolerance test result if applicable
Three readings

Reading (1)……… mmol/L
Reading (2)…… …mmol/L
Reading (3)…… …mmol/L

26. HBA1C if applicable ……………………%

E-History of previous pregnancies and deliveries

27. Have you been **diagnosed** with any of these health problems during your **<u>previous pregnancies</u>**?(Plus medical records will be reviewed)<br><br>***Single or multiple choices could be selected if applicable.***	☐ Gestational Diabetes<br>☐ Baby weight equal or more than 4kg<br>☐ Pregnancy induced Hypertension<br>☐ Eclampsia<br>☐ Hyperemesis Gravidarium<br>☐ Urinary tract infection<br>☐ Thyroid disorders<br>☐ Anemia<br>☐ Pregnancy related Anxiety<br>☐ Antenatal Depression<br>☐ Schizophrenia<br>☐ Bipolar disorder<br>☐ Panic attacks<br>☐ Abnormal ultrasound for embryo<br>☐ Abnormal ultrasound for placenta or amniotic fluids<br>☐ None<br>☐ Others please specify…..
28. Did you face any complications related to your **<u>previous deliveries</u>**?(Plus medical records will be reviewed)<br><br>***Single or multiple choices could be selected if applicable.***	☐ Post-partum bleeding<br>☐ Infection<br>☐ Preterm labor (before 37 weeks)<br>☐ Instrumental delivery<br>☐ Emergency C-Section<br>☐ Planned C –Section<br>☐ Postpartum Depression<br>☐ Complications related to newborn<br>☐ Others please specify…<br>☐ None

F-Chronic (before pregnancy)

29. **<u>Before your current pregnancy</u>**: Did you have been diagnosed of any of these medical conditions?(Plus medical records will be reviewed)<br><br>***Single or multiple choices could be selected if applicable.***	☐ High Blood Pressure<br>☐ Heart problems<br>☐ Diabetes type I<br>☐ Diabetes type II<br>☐ Thyroid problem<br>☐ Anemia<br>☐ Kidney diseases<br>☐ Asthma<br>☐ Cancer<br>☐ Others…

Institutional Review Board · Hamad Medical Corporation · Doha · Qatar
حمد Hamad

30. Kindly specify duration of the selected illness?(illness 1) ……………(months)

31. Kindly specify duration of the selected illness?(illness 2) ……………(months)

I-Medications History

32. **<u>In your current pregnancy</u>**: What medications are you taking?

    Single or multiple choices could be selected if applicable

    - ☐ Multivitamins
    - ☐ Hypoglycemic medication such as :Glucophage
    - ☐ Insulin injections
    - ☐ Thyroid supplement
    - ☐ Antacids
    - ☐ Anti-depressant
    - ☐ Anti-anxiety
    - ☐ Others specify…
    - ☐ None

33. If your taking medications related to mood disorders (anxiety and depression) are you taking it regularly (as prescribed)?
    - ☐ (0)No,
    - ☐ (1)Yes

34. What are the reasons behind not taking your medications regularly?
    - ☐ (1)I am pregnant and I am scared it will affect baby.
    - ☐ (0)My physician stopped it.
    - ☐ Others please specify….

J-Anthropometric measures based on the first visit

35. Pre-pregnancy BMI(kg/m^2) (first visit BMI)

    Weight……………(kg)
    Height ………….. (m^2).
    BMI…………..(Kg/m^2)

K-Life style habits

36. Do you do regular sport different from your house activity?
    - ☐ (1)No
    - ☐ (0)Yes

37. How much time you spend on sport activities?

- ☐ (0)<20 min
- ☐ (1)>20min

38. How many times per week you do sport?

- ☐ (0)< 1 time/wk.
- ☐ (1)Equal to 1 to 2 time/wk.
- ☐ (2)> 2 time/wk.

39. Interpretation of Fitness Score for question 52 to 55:

- ☐ ***<u>Not fit</u>*****:** perform physical activity less than once or twice a week and for a period of time of less than 20 minutes (zero index score)
- ☐ ***<u>Active</u>***: perform physical activity once to twice a week for 20 minutes, or more than twice a week for less than 20 minutes have a physical activity index of one
- ☐ ***<u>Fit</u>***: perform physical activity more than twice a week for more than 20 minutes are considered to be fit

40. Do you smoke?

- ☐ Never smoked
- ☐ Former smoker(*smoked at least 100 cigarettes but currently not smoking*)
- ☐ Current smoker
- ☐ Every day smoker

41. Do you drink alcohol?

- ☐ No, never had alcohol before.
- ☐ I don’t drink now but I used to drink before pregnancy.
- ☐ Yes, I am drinking alcohol during my pregnancy.

MRC-01-18-188 Validity: 08 05 2019 - 06 05 2020 E-stamped 12 Jun 2019
